# *SFRP4* protein expression is reduced in high grade astrocytomas which is not caused by the methylation of its promoter

**DOI:** 10.3389/fnmol.2024.1398872

**Published:** 2024-06-27

**Authors:** Anja Kafka, Nives Pećina-Šlaus, Denis Drmić, Anja Bukovac, Niko Njirić, Kamelija Žarković, Antonia Jakovčević

**Affiliations:** ^1^Laboratory of Neuro-oncology, Croatian Institute for Brain Research, School of Medicine, University of Zagreb, Zagreb, Croatia; ^2^Department of Biology, School of Medicine, University of Zagreb, Zagreb, Croatia; ^3^Department of Neurosurgery, University Hospital Center “Zagreb”, School of Medicine, University of Zagreb, Zagreb, Croatia; ^4^Department of Pathology, School of Medicine, University of Zagreb, Zagreb, Croatia; ^5^Division of Pathology, University Hospital Center “Zagreb”, Zagreb, Croatia

**Keywords:** astrocytoma, Wnt signaling pathway, SFRP4, promoter methylation, methylation specific PCR, IRS score

## Abstract

**Introduction:**

Epigenetics play a vital role in stratifying CNS tumors and gliomas. The importance of studying Secreted frizzled-related protein 4 (SFRP4) in gliomas is to improve diffuse glioma methylation profiling. Here we examined the methylation status of *SFRP4* promoter and the level of its protein expression in diffuse gliomas WHO grades 2–4.

**Methods:**

SFRP4 expression was detected by immunohistochemistry and evaluated semi-quantitatively. In the tumor hot-spot area, the intensity of protein expression in 200 cells was determined using ImageJ (National Institutes of Health, United States). The assessment of immunopositivity was based on the IRS score (Immunoreactivity Score). Promoter methylation was examined by methylation specific-PCR (MSP) in fifty-one diffuse glioma samples and appropriate controls. Isolated DNA was treated with bisulfite conversion and afterwards used for MSP. Public databases (cBioPortal, COSMIC and LOVD) were searched to corroborate the results.

**Results and discussion:**

SFRP4 protein expression in glioblastomas was very weak or non-existent in 86.7% of samples, moderate in 13.3%, while strong expression was not observed. The increase in astrocytoma grade resulted in SFRP4 protein decrease (*p* = 0.008), indicating the loss of its antagonistic role in Wnt signaling. Promoter methylation of *SFRP4* gene was found in 16.3% of cases. Astrocytomas grade 2 had significantly more methylated cases compared to grade 3 astrocytomas (*p* = 0.004) and glioblastomas (*p* < 0.001), which may indicate temporal niche of methylation in grade 2. Furthermore, the expression levels of SFRP4 were high in samples with methylated *SFRP4* promoter and low or missing in unmethylated cases (Pearson’s R = −0.413; *p* = 0.003). We also investigated the association of SFRP4 changes to key Wnt regulators *GSK3β* and *DKK3* and established a positive correlation between methylations of *SFRP4* and *GSK3β* (Pearson’s R = 0.323; *p* = 0.03). Furthermore, SFRP4 expression was correlated to unmethylated *DKK3* (Chi square = 7.254; *p* = 0.027) indication that Wnt signaling antagonist is associated to negative regulator’s demethylation.

**Conclusion:**

The study contributes to the recognition of the significance of epigenetic changes in diffuse glioma indicating that restoring SFRP4 protein holds potential as therapeutic avenue. Reduced expression of SFRP4 in glioblastomas, not following promoter methylation pattern, suggests another mechanism, possible global methylation, that turns off SFRP4 expression in higher grades.

## Introduction

Gliomas comprise about 30 percent of all brain tumors and about 80 percent of all malignant brain tumors. Currently histological and molecular features are strongly integrated in glioma classification (Louis et al., 2021). The most comprehensive changes were figured out for different glioma types and now four general groups of diffuse gliomas are recognized. Astrocytomas are the most common type characterized by diffuse infiltrative growth in the brain parenchyma. WHO tumor grading is essential in risk stratification. The inclusion of both phenotypic and molecular parameters has led to changes in the 2016 WHO (World Health Organization) classification (Louis et al., 2016) and improved the prognosis of gliomas (Louis et al., 2021). Now all diffuse gliomas, whether astrocytic or not, are grouped into one category based on mitotic activity, diffuse growth pattern, and the mutational status of the *IDH1* and *IDH2* genes, together with several other molecular biomarker testing. Tumors are graded within tumor types rather than across different types (Park et al., 2023). For example, patients with grade 3 gliomas carrying a 1p/19q co-deletion have a better prognosis than IDH wild-type grade 2 glioma. The prognosis of diffuse glioma depends on several factors including tumor grade. In general, grade 2 tumors have 8 year overall median survival while patients with grade 3 tumor are living 2–5 years after diagnosis. Despite recent advances in diagnosis and treatment of glioblastoma, the prognosis of the disease is poor. Glioblastoma have a very high mortality rate, with average survival time from 12 to 18 months (Barthel et al., 2018; Narayanan and Turcan, 2020; Barthel et al., 2022; Malta et al., 2024).

Previously, it has been demonstrated that specific family of WNT signaling antagonists - the Secreted Frizzled-Related Protein (SFRP) family, is responsible for the decrease in proliferation of glioma cells and for making them sensitive to chemotherapeutics (Warrier et al., 2013; Schiefer et al., 2014). The SFRPs have a far-reaching effect across Wnt pathways, being able to antagonize both canonical and noncanonical branches (Yu et al., 2019). The canonical pathway is regulated at multiple levels. A fine-tuned homeostatic balance between cytoplasmic and nuclear β-catenin determines the final outcome of the Wnt signalization. The balance is achieved by extracellular antagonists of Wnt signaling (Schiefer et al., 2014). They are expressed in various tissues and control a multitude of biological processes during embryonic development and adulthood (Nusse and Clevers, 2017; Rim et al., 2022). In the absence of the Wnt ligand, a β-catenin destruction complex is formed in the cytoplasm. Phosphokinases CK1 and GSK3β sequentially phosphorylate axin-bound β-catenin on a series of regularly spaced serine and threonine residues at its N-terminus. Phosphorylated motifs act as a dock for the E3 ubiquitin ligase subunit β-TrCP (β-Transducin Repeat Containing Protein), which induces ubiquitination and consequently proteasomal degradation of β-catenin. Thus, the level of β-catenin in the cell remains low (Hayat et al., 2022; Rim et al., 2022). Binding of Wnt ligand to membranous receptor Frizzled (Fz) and its co-receptor low density lipoprotein receptor related protein (LRP) initiates a cascade of events leading to the disassembly of the APC/Axin/GSK3β/CK1 destruction complex and the stabilization of cytoplasmic β-catenin. Accumulation of β-catenin in the cytoplasm leads to its translocation to the nucleus and activation of target genes mediated by transcription factors TCF/LEF (Hayat et al., 2022; Rim et al., 2022). In recent years, Wnt antagonists received a lot of attention due to their frequent inactivation in cancer (Pawar and Rao, 2018). *SFRP* genes (in human number 1–5) are frequently hypermethylated in a variety of human cancers and thereby transcriptionally silent (Schiefer et al., 2014). Silencing of *SFRP* genes via hypermethylation at the promoter region has been reported for glioblastoma, too (Götze et al., 2010; Schiefer et al., 2014; Bhuvanalakshmi et al., 2018). Suppression of Wnt signaling by SFRP proteins also contributes to normal astrocyte development (Sun et al., 2015).

Secreted frizzled-related protein 4 (SFRP4) is an extracellular modulator of the Wnt signaling that can bind both the Wnt ligand and frizzled receptor. Thus, it acts as an antagonist of Wnt ligands, preventing their binding to the receptor. With a molecular weight of 39.9 kDa and a length of 346 amino acids, SFRP4 is the largest member of the SFRP family (Pohl et al., 2015). Altered SFRP4 expression in different types of tumors indicates its essential role in maintaining tissue homeostasis (Bovolenta et al., 2008; Pawar and Rao, 2018). Also, silenced *SFRP4* gene or its decreased protein expression causes the activation of the Wnt pathway. *SFRP4* contains dense CpG islands flanking the first exon. Their hypermethylation is one of the mechanisms of gene silencing, which creates a predisposition for malignant changes (Müller and Győrffy, 2022; Ramazi et al., 2023).

Whilst the aforementioned studies have shown that SFRP4 could be involved in the molecular pathogenesis of diffuse gliomas, its specific role in astrocytoma subtypes has not been sufficiently investigated. Therefore, the present study aims to explore the *SFRP4* promoter methylation status and its consequence on protein levels in diffuse astrocytoma grade 2–4. In order to elucidate *SFRP4’s* effect on Wnt cascade its correlation to GSK3β, DKK1, DKK3, LEF1 and β-catenin was also tested in our study.

## Materials and methods

### Tissue samples

Fifty-one diffuse astrocytoma sample graded from 2 to 4 together with paired blood and formalin-fixed paraffin-embedded (FFPE) slides of tumor tissues were collected from the Department of Neurosurgery and Department of Pathology University Hospital Center “Zagreb.” Certified neuropathologist (KŽ, AJ) reviewed chosen slides to set the accurate diagnosis (CNS WHO grade 2–4) in concordance with the most recent WHO classification (Louis et al., 2021). The patients included in the study had no family history of brain tumors and did not undergo any cancer treatment, prior to surgery, which could affect the results of molecular analyses. The sample consisted of 11 astrocytoma CNS WHO grade 2, 10 astrocytoma CNS WHO grade 3 and 30 astrocytoma CNS WHO grade 4. Twenty-nine patients were male and twenty-two were female. The age of patients varied from 6 to 83 (mean age = 50.31, median = 54 years, std. deviation = 18.099). The mean age of diagnosis for males was 47 years (median 49, std. deviation = 19.085) and for females 56 (median 63, std. deviation = 15.710) (Table 1).

**Table 1:** Demographic and clinical data of astrocytoma patients.

Patient no	Grade	Age	Sex	Molecular features
1	2	31	M	IDH1 +, ATRX+
2	2	39	F	IDH1 +,1p19q codeletion positive, ATRX-, p53+
3	2	31	F	IDH1 +, ATRX+, p53+
4	2	36	F	IDH1 +
5	2	32	M	IDH1 +,1p19q codeletion positive, ATRX-, p53+
6	2	49	M	IDH1 +, ATRX+, p53+
7	2	27	M	IDH1 -, ATRX+, p53-
8	2	44	M	IDH1 +, ATRX+, p53+
9	2	56	M	IDH1 +
10	2	38	F	IDH1 +
11	2	48	M	IDH1 +
12	3	35	M	ND
13	3	66	F	IDH1 -, ATRX -, p53+
14	3	24	M	IDH1 -, NOS, p53+
15	3	34	M	IDH1 +, ATRX+, p53+
16	3	29	M	IDH1 +, ATRX-
17	3	51	M	IDH1 -
18	3	34	F	IDH1 +, ATRX+, p53+
19	3	55	F	IDH1 +
20	3	58	M	IDH1+
21	3	46	M	IDH1+
22	4	67	F	ND
23	4	68	M	IDH1 -
24	4	62	M	ND
25	4	77	F	IDH1 -
26	4	61	F	ND
27	4	40	F	IDH1 +
28	4	65	F	IDH1 -
29	4	30	M	IDH1 +
30	4	58	M	IDH1 -
31	4	77	F	P53+
32	4	42	M	IDH1 -, P53+
33	4	54	M	IDH1 -, ATRX-, P53+
34	4	65	F	IDH1 -, 1p19q codeletion negative, ATRX-, p53+
35	4	71	F	IDH1 -, 1p19q codeletion negative, ATRX-, p53+
36	4	83	M	IDH1 +
37	4	68	M	IDH1 -
38	4	55	F	ND
39	4	54	M	IDH1 -, 1p19q codeletion negative, ATRX-, p53+
40	4	39	F	IDH1 -, ATRX+, p53+
41	4	56	M	IDH1 -, 1p19q codeletion negative, ATRX+, p53+
42	4	70	F	ND
43	4	62	M	IDH1 -, p53+
44	4	53	F	IDH1 -
45	4	67	F	IDH1 -, p53+
46	4	79	M	IDH1 -
47	4	72	F	IDH1 -
48	4	6	M	IDH1 -
49	4	6	M	IDH1 -
50	4	69	M	ND
51	4	65	F	IDH1 -, p53+

M, male; F, female; IDH1 + , IDH1 mutant; IDH1 -, IDH1 wild-type; ATRX + , strong nuclear postivity; ATRX -, loss of ATRX expression (mutated); p53 + , p53 mutation.

The study was approved by the Ethical Committees, School of Medicine University of Zagreb (Case number: 380–59–10,106-14-55/147; Class: 641–01/14–02/01) and University Hospital Center “Zagreb” (number 02/21/JG, class: 8.1.-14/54–2). Patients gave their informed consent.

### Immunohistochemistry (IHC)

IHC staining was performed on 4 μm thick FFPE sections mounted on silanized glass slides (DakoCytomation, Glostrup, Denmark). Tissue sections went through deparaffinization in xylene (3x, 5 min), rehydration in graded ethanol series, (100, 96 and 70% ethanol, 2x, 3 min), and water (30 s). Next, sections were heated in 6 M citrate buffer in the microwave oven two times for 10 min at 400 W and three times for 5 min at 350 W in order to recover antigen epitopes. Afterward, the endogenous peroxidase activity was blocked using 3% hydrogen peroxide for 10 min in dark. Non-specific binding was blocked by incubating samples with protein block serum-free ready-to-use (Agilent Technologies, United States) for 30 min at 4°C. Sections were incubated with primary antibody Anti-SFRP-4 [EPR9389] (rabbit monoclonal anti-human; ab154167, Abcam, United States; dilution 1:100) overnight at 4°C. Dako REAL Envision detection system Peroxidase/DAB, Rabbit/Mouse, HRP (Agilent Technologies, United States) was used for visualization following the manufacturer’s instructions and the sections were afterwards counterstained with hematoxylin.

The level of SFRP4 expression in the healthy brain was determined by using the cerebral cortex of the human brain (Amsbio, Oxfordshire, UK). The level of immunoreactivity in the healthy brain tissue was moderate, and the signal was detected in the cytoplasm. Human ovarian carcinoma tissue that, according to the antibody datasheet, expresses SFRP4 was used as positive control. Negative controls underwent the same procedure with the omission of incubation with primary antibody.

### Semiquantitative analysis by IRS score

Tissue sections were examined using Olympus BX52 microscope (Olympus Life Science). In the tumor hot-spot area, 200 cells were counted and the intensity of protein expression was determined using the computer program ImageJ (National Institutes of Health, United States). The assessment of immunopositivity in the membrane, cytoplasm and/or nuclei of tumor cells was based on the determination of the staining IRS score (Immunoreactivity Score). IRS is the number obtained by multiplying the percentage of cells with positive signal (PP, Positive Cells Proportion Score) with the intensity of the signal (SI, Staining Intensity Score). Five different categories of staining power (PP) were determined: (0) no immunopositivity in tumor cells, (1) immunopositivity in 1–25% of tumor cells, (2) immunopositivity in 26–50% of tumor cells, (3) immunopositivity in 51–85% of tumor cells, (4) immunopositivity in >85% of tumor cells. The staining intensity (SI) was assessed into three categories: (1) no/weak immunopositivity-yellowish staining, (2) moderate-brownish staining, (3) strong-dark brown staining. Due to the needs of statistical analysis, IRS values ranging from 0 to 12 were assessed: 1 (IRS = 0–4) no expression or weak expression, 2 (IRS = 5–8) moderate expression and 3 (IRS = 9–12) strong expression.

### DNA extraction

The genomic DNA extraction from unfixed frozen tumor tissue was performed according to the protocol by Green and Sambrook (1989). Briefly, approximately 0.5 g of tumor tissue was homogenized with 1 mL extraction buffer (10 mM Tris–HCl, pH 8.0; 0.1 M EDTA, pH 8.0; 0.5% sodium dodecyl sulfate) and incubated with proteinase K (100 μg/mL; Sigma-Aldrich, St. Louis, MO, United States) overnight at 37°C. Organic (phenol–chloroform) extraction and ethanol precipitation followed. The extracted DNA was successfully used for epigenetic (MS-PCR) analysis.

### Methylation-specific PCR (MSP)

Isolated DNA was treated with bisulfite using the MethylEdge Bisulfite Conversion System (Promega, Madison, Wisconsin, United States) following the manufacturer’s instruction. Bisulfite-treated DNA was afterward used for methylation-specific PCR (MSP). Primer sequences for *SFRP4* promoter region for MSP were synthesized according to Schiefer et al. (2014): methylated primers, F: 5’ GGGTGATGTTATCGTTTTTGTATCGAC 3′ and R: 5’ CCTCCCCTAACGTAAACTCGAAACG 3′; unmethylated primers, F: GGGGGTGATGTTATTGTTTTTGTATTGAT and R: CACCTCCCCTAACATAAACTCAAAACA 3′. Expected product size for methylated reaction was 111 bp, and for unmethylated reaction 115 bp.

PCRs for bisulfite-treated DNA were performed using TaKaRa EpiTaq HS (TaKaRa Bio, Unites States): 1XEpiTaq PCR Buffer (Mg2+ free), 2.5 mM MgCl2, 0.3 mM dNTPs, 20 pmol of each primer (Sigma-Aldrich, USA), 50 ng of DNA, and 1.5 Units of TaKaRa EpiTaq HS DNA Polymerase in a 25 μL final reaction volume. PCR cycling conditions were as following: initial denaturation at 95°C for 5 min, followed by 35 cycles consisting of three steps: 95°C for 30 s, the respective annealing temperature for 30 s, 72°C for 30 s, followed by a final extension at 72°C for 7 min. For the amplification of methylated *SFRP4* promoter region the annealing temperature was 65°C, while for unmethylated *SFRP4* promoter region was 63°C.

PCR products were separated on 2% agarose gel stained with Syber Safe nucleic acid stain (Invtrogen, Thermo Scientific, United States) and visualized on a UV transilluminator. Methylated Human Control (Promega, Madison, Wisconsin, United States) was used as a positive control for the methylated reaction, while unmethylated DNA EpiTect Control DNA (Qiagen, Hilden, Germany) served as a positive control for the unmethylated reaction. Nuclease-free water was used as a negative control.

Samples that displayed bands in methylated reactions were classified as methylated. In our experiment, all methylated samples also showed parallel unmethylated bands for specific patient denoting unmethylated promoters. However, due to the appearance of methylated promoters, we classified those samples as methylated since amplification of a band is observed in methylated reaction. The presence of both reactions, methylated and unmethylated, can be explained by glioma’s intrinsic intra-tumor heterogeneity, so some tumor cells will have unmethylated promoters and others methylated. We should also consider point of time in this process where methylation or unmethylation can happen in specific time frame. The other possibility is that there might be DNA extracted from non-tumor origin from abundance of cells that form tumor mass that may be amplified to show parallel unmethylated bands. We can discuss the band intensities as possible cut-off point for methylation, but we decided to classify binary when band is present in the methylated reaction the promoter was classified as methylated.

### Statistical analysis

Methylation status of *SFRP4* gene, data on its protein expression levels were analyzed together with grade, and other clinical and demographic features. Statistical analysis was performed using SPSS v.19.0.1 (SPSS, Chicago, IL, United States) statistical program. The significance level was set at *p* < 0.05.

The normality of the distribution of the obtained data was assessed with the Kolmogorov–Smirnov test. A *p*-value lower than *p* < 0.05 indicates that the distribution is significantly different from normal. In the case of normal distribution, differences in the values between astrocytoma grades were examined using one-way analysis of variance (ANOVA), and in case of deviation from normality, the Kruskal-Wallis test was used. Differences in values between the two groups were tested with Student’s t-test in case of normal distribution, and in case of deviation from normality with Mann–Whitney test. Differences in the frequency of the analyzed features were tested with the Pearson χ2 test.

Pearson and Spearman’s correlations were used to test the relationships between SFRP4 and GSK3β, DKK1, DKK3, LEF1 and β-catenin.

### Analysis of public database cBioPortal and LOVD

In order to test the compatibility of our results we investigated data from publicly available databases. Genetic changes reported on *SFRP4* gene were assessed from cBioPortal (https://www.cbioportal.org/, accessed on 29^th^ February 2024) (Cerami et al., 2012), COSMIC^1^ and LOVD (Leiden Open Variation Database, https://databases.lovd.nl/shared/gene), a publicly available databases for tumor genomics and transcriptomics. The *in silico* analysis was performed on the combined study encompassing 3,735 samples. The analysis queried following studies: Brain Lower Grade Glioma (TCGA, Firehose Legacy); Diffuse Glioma (GLASS Consortium); Diffuse Glioma (GLASS Consortium, Nature 2019); Diffuse Glioma (MSK, Clin Cancer Res 2024); Glioma (MSK, Clin Cancer Res 2019); Glioma (MSK, Nature 2019); Low-Grade Gliomas (UCSF, Science 2014); Brain Tumor PDXs (Mayo Clinic, Clin Cancer Res 2020); Glioblastoma (Columbia, Nat Med. 2019); Glioblastoma Multiforme (TCGA, PanCancer Atlas); Glioblastoma (CPTAC, Cell 2021).

## Results

### Tumor tissue samples

Analysis of SFRP4 protein expression and *SFRP4* promoter methylation status was performed on a total of 51 patients. Regarding tumor grade, there were 11 samples of diffuse astrocytoma (21.6%), 10 samples of anaplastic astrocytoma (19.6%) and 30 samples of glioblastoma (58.8%). Results of the ANOVA test showed significant differences between patient age and astrocytoma grade (*p* = 0.002). Glioblastomas were found to occur later in life compared to grade 2 (*p* = 0.003) and grade 3 astrocytoma (*p* = 0.027).

### SFRP4 protein expression in astrocytoma samples

**Figure 1:**
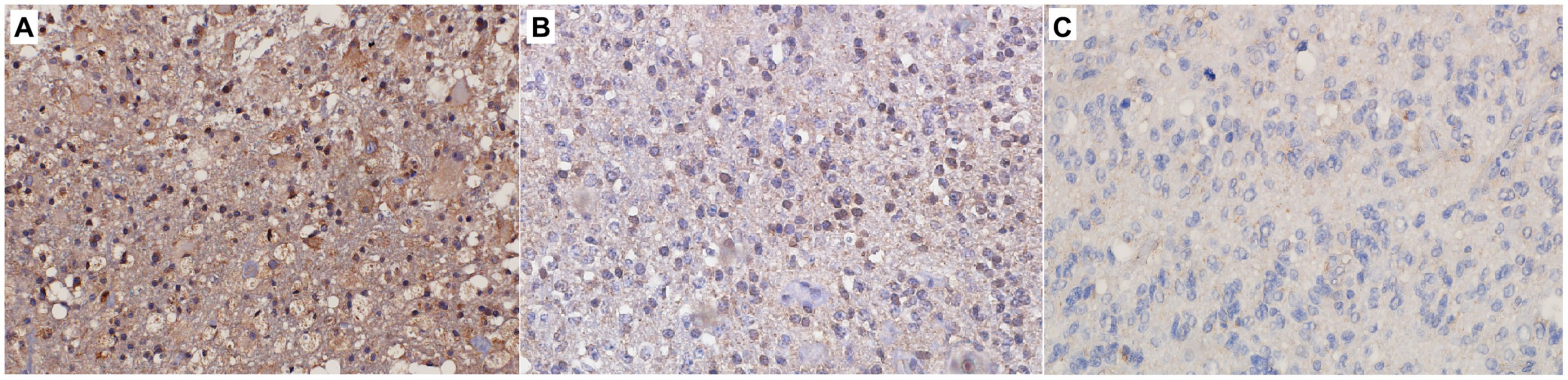
Immunohistochemical staining of **(A)** CNS WHO grade 2 astrocytoma, **(B)** CNS WHO grade 3 astrocytoma, **(C)** CNS WHO grade 4 astrocytoma. Figure shows strong **(A)**, moderate **(B)** and weak **(C)** expression of the SFRP4 protein (200x magnification).

**Figure 2:**
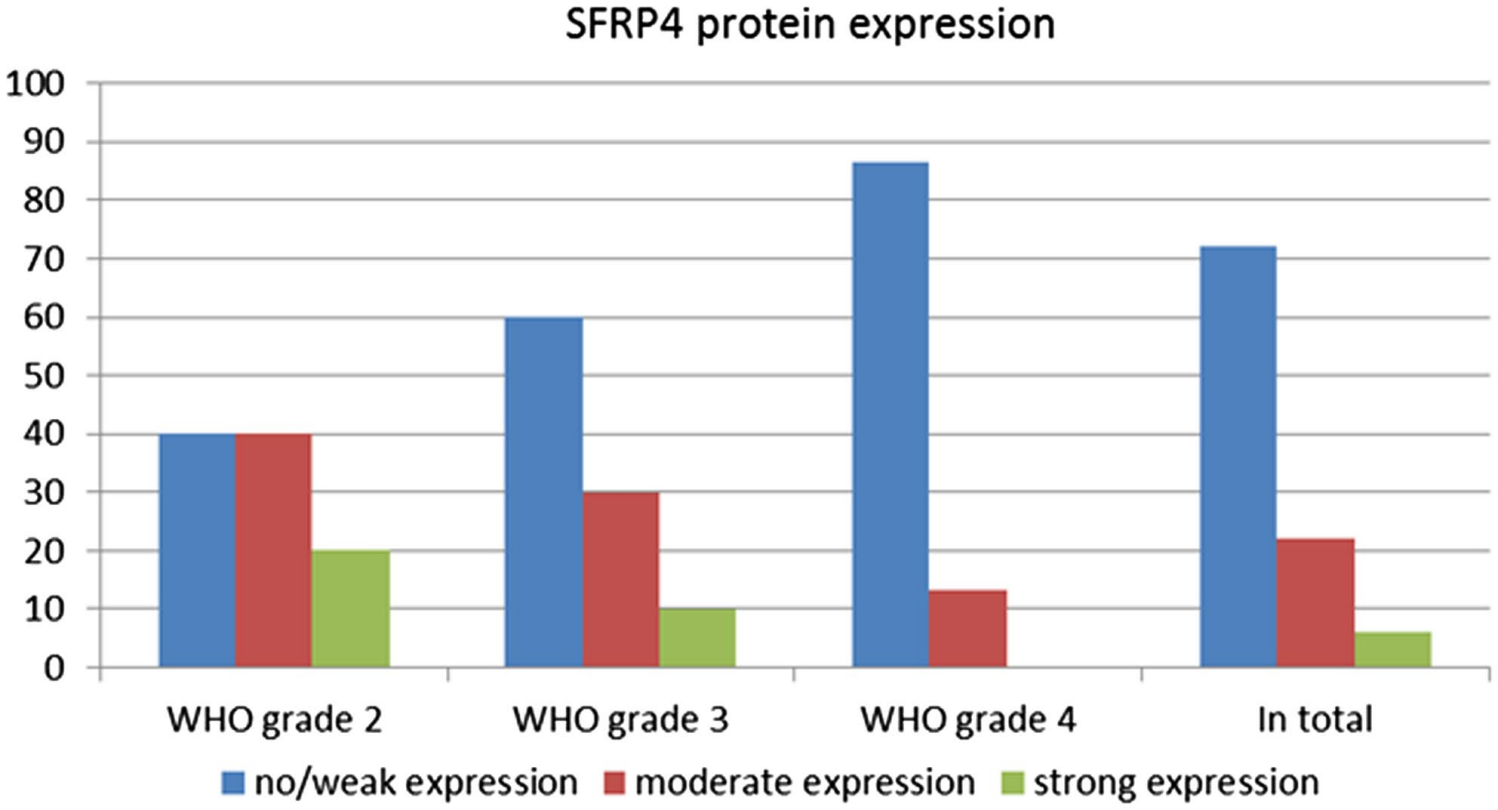
SFRP4 protein expression between different astrocytoma grades.

The levels of SFRP4 protein were generally low in our total sample. SFRP4 expression in a total of 50 astrocytomas of different grades showed low or lack of expression in 72% (36/50), moderate in 22% (11/50) and strong in 6% (3/50) of cases. The Kruscal-Wallis test revealed a significant difference in the expression level of SFRP4 protein regarding tumor grade (*p* = 0.008), and the Spearman test also confirmed a moderate negative correlation between the analyzed variables (rs = −0.442, *p* = 0.001). A significantly higher number of samples with moderate and strong expression was present in the group of diffuse astrocytomas (CNS WHO grade 2), while samples with low protein expression predominated in higher astrocytoma grades (CNS WHO grades 3 and 4) (*p* = 0.002). Diffuse astrocytomas showed low or no expression in 40% of samples, moderate in 40%, while 20% of samples had strong expression. In the group of anaplastic astrocytomas, 60% of samples showed low or no expression, 30% had moderate, while 10% showed strong expression. SFRP4 protein expression in glioblastomas was very weak or non-existent in 86.7%, and moderate in 13.3% of samples, while strong expression was not observed in glioblastoma (Table 2; Figure 1). The signal was localized on the membrane, in the cytoplasm and in the nucleus (Figure 2). All the samples showed immunopositivity in the cytoplasm, 34.7% of samples has signal also in the nucleus and membrane in addition to the cytoplasm and 14.3% of samples has signal in the cytoplasm and membrane but not in the nucleus. However, we did not observe that localization of *SFRP4* was associated with an aggressive phenotype or survival. We also checked if *SFRP4* localization had any association with all other investigated variables but could not establish such connection (Table 3).

**Table 2:** SFRP4 protein expression in different astrocytoma grades.

IRS value		SFRP4		
		Grade 2	Grade 3	Grade 4
IRS 0–4	N	4/10	6/10	26/30
	%	40	60	86.7
IRS 6–8	N	4/10	3/10	4/30
	%	40	30	13.3
IRS 9–12	N	2/10	1/10	0/30
	%	20	10	0
In total	N	10	10	30
	%	100	100	100

N, number of samples; %, percentage of samples.

**Table 3:** Promoter methylation status, expression levels and localizations of Secreted Frizzled Related Protein 4 (SFRP4) protein in astrocytoma samples and patients’ survival.

Patient no	*SFRP4* methylation	*SFRP4* expression	Localization	Survival (months)
1	U	2	C	103
2	M	2	C + M + N	61
3	M	1	C	71
4	U	1	C + M + N	105
5	U	1	C	129
6	M	3	C + M + N	42
7	M	3	C + M + N	20
8	M	ND	ND	79
9	M	2	C + M	ND
10	M	1	C + M + N	ND
11	M	2	C + M	ND
12	U	1	C	ND
13	ND	1	C + M + N	4
14	U	2	C + M + N	83
15	U	1	C + M + N	55
16	U	1	C + M + N	100
17	U	2	C + M + N	14
18	U	1	C	ND
19	U	2	C	ND
20	U	3	C + M	ND
21	U	1	C + M + N	ND
22	U	1	C + M	7
23	U	1	C	10
24	U	2	C	18
25	U	1	C + M + N	26
26	U	1	0	10
27	U	1	C	25
28	U	1	C	47
29	U	1	0	81
30	U	1	C	11
31	U	1	C	ND
32	U	1	C	48
33	U	1	C	19
34	U	1	C + M	5
35	U	2	C + M	13
36	U	1	C	33
37	U	1	C + M + N	6
38	U	2	C + M + N	97
39	U	1	C	10
40	U	1	C + M + N	41
41	U	1	C	25
42	U	1	C	ND
43	U	1	C	36
44	U	1	C + M	5
45	ND	1	C + M + N	ND
46	U	1	C	3
47	U	2	C	3
48	U	1	C	ND
49	U	1	C + N	10
50	U	1	C + M + N	8
51	U	1	C	14

M, methylated; U, unmethylated; 1, weak or lack of expression; 2, moderate expression; 3, strong expression; C, cytoplasmic; M, membranous; N, nuclear; ND, not determined.

### Methylation status of the *SFRP4* gene promoter

**Figure 3:**
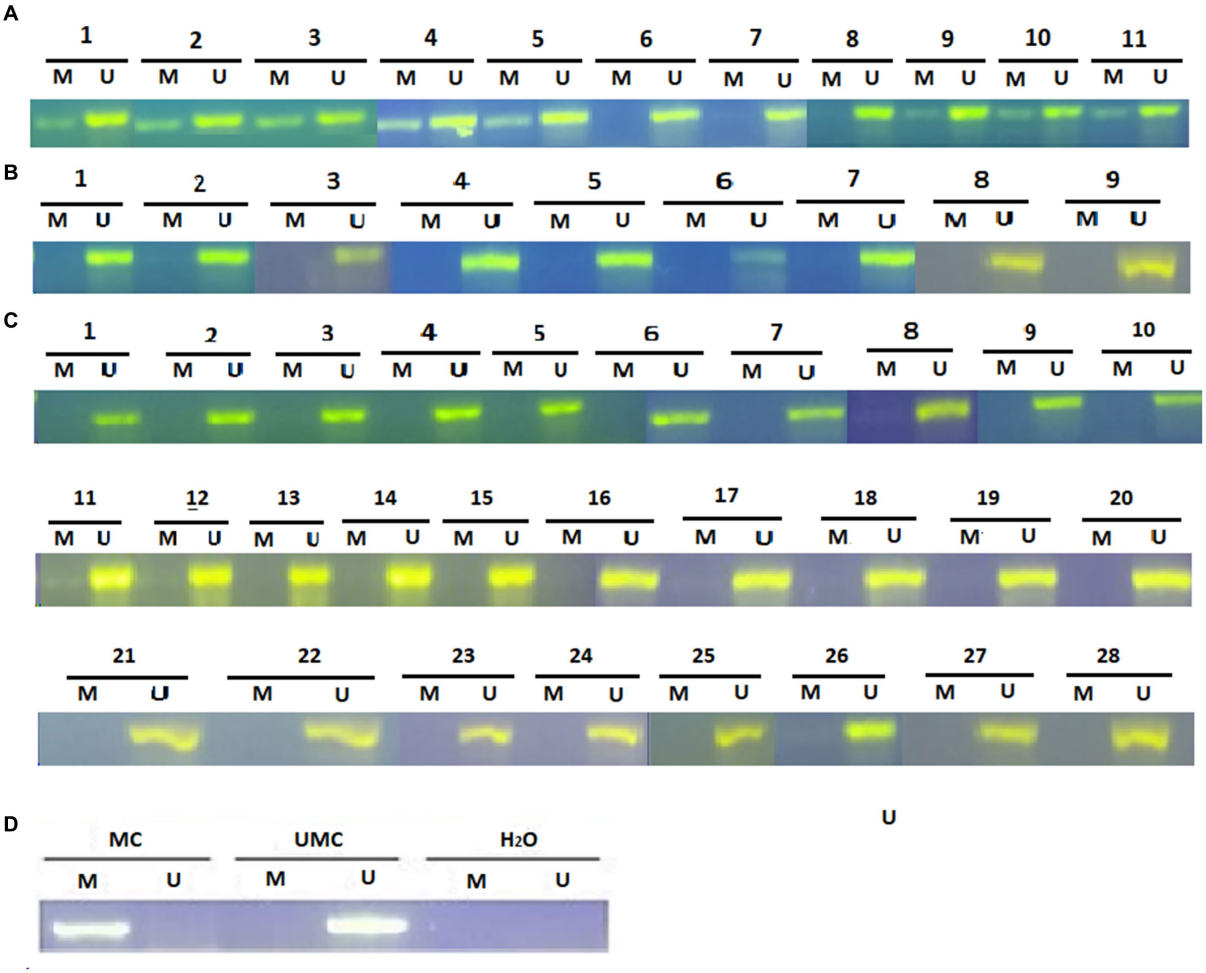
Methylation-specific PCR (MSP) analysis for *SFRP4* gene promoter in astrocytic brain tumors **(A)** grade 2, **(B)** grade 3 and **(C)** grade 4. The presence of a visible PCR product in lanes marked U indicates the presence of unmethylated promoters; the presence of a product in lanes marked M indicates the presence of methylated promoters. **(D)** Methylated human control (MC) was used as positive control for methylated reaction, unmethylated human control (UMC) was used as positive control for unmethylated reaction, and water served as negative control. M, methylated reaction; UM, unmethylated reaction.

Promoter methylation of the *SFRP4* gene in astrocytoma samples was examined by the MS-PCR. The detected product was between 100 and 150 bp long. The MS-PCR reaction was considered optimized when the methylated control sample gave the product of the desired size only in the methylated reaction of the methylated control while the unmethylated control sample gave product in the unmethylated reaction of the unmethylated control. Nuclease-free water was used as a negative control and the absence of product formation in it was evidence that no contamination was present. In case the product was formed only in the unmethylated reaction, the sample was considered unmethylated. In case the product was formed in both, the methylated and the unmethylated reaction, the sample was considered methylated. Results of the reaction for each sample are shown in Figure 3.

**Figure 4:**
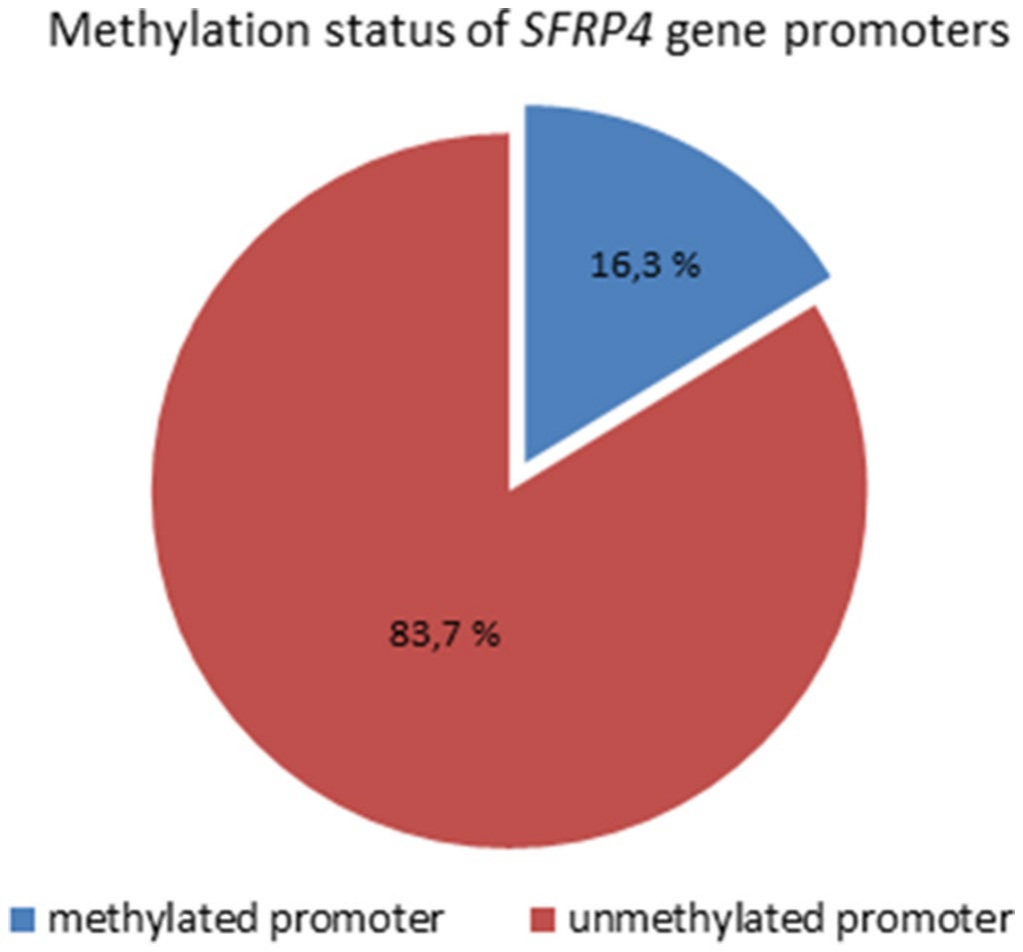
Methylation status of SFRP4 gene promoters in total astrocytoma samples of different grades.

**Figure 5:**
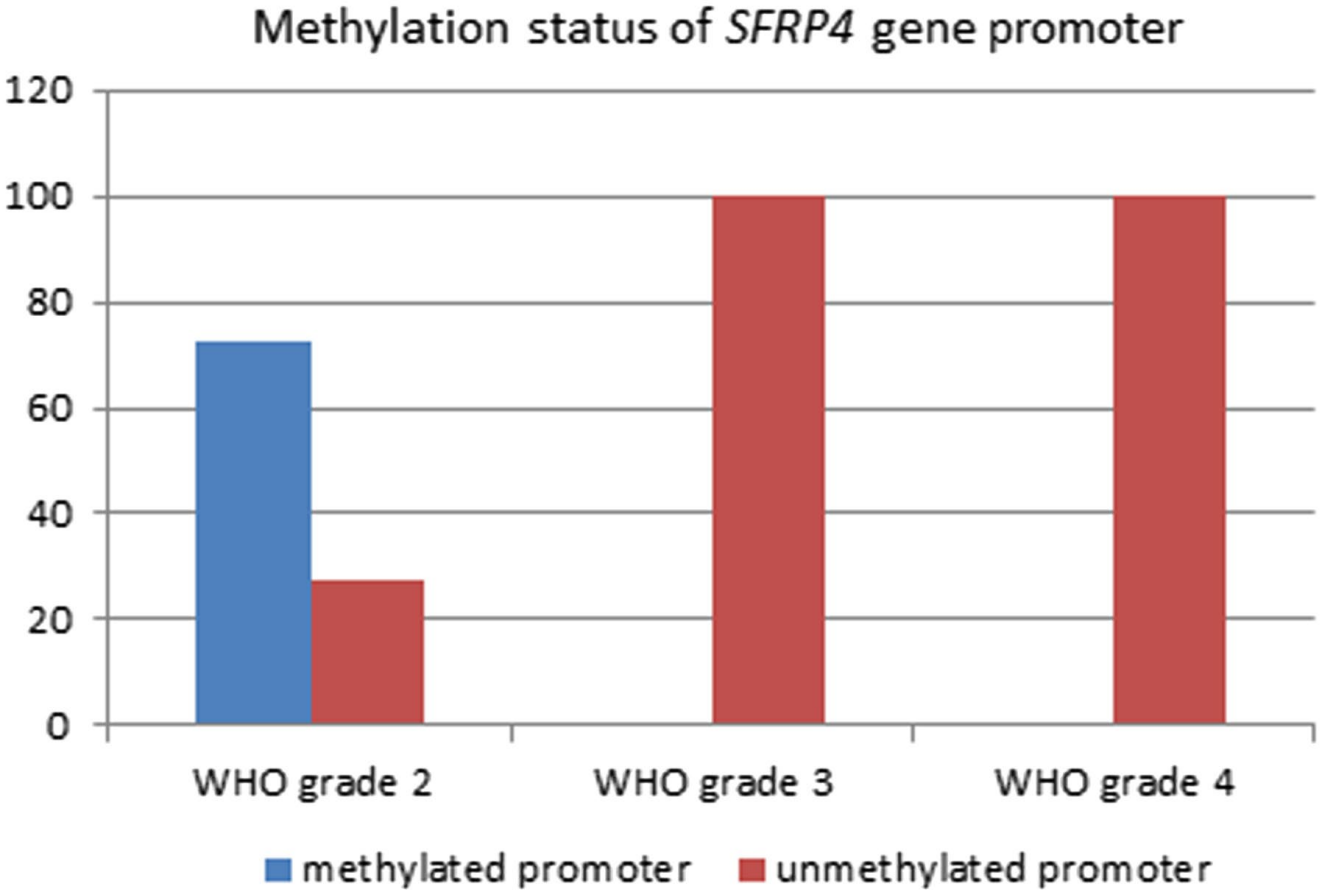
Percentage of SFRP4 promoter methylation across different astrocytoma grades.

Analysis of 49 astrocytoma samples showed that 16.3% (8/49) of cases had a methylated *SFRP4* promoter while 83.7% (41/49) had an unmethylated one (Figure 4). DNA promoter methylation of *SFRP4* was exclusively observed in diffuse astrocytoma (8/11 cases, 72.7%), while all anaplastic astrocytomas and glioblastomas samples were unmethylated (Figure 5). Pearson’s χ2 - test showed statistically significant differences in methylation status of the *SFRP4* gene between astrocytoma malignancy grades (*p* < 0.001). *Post-hoc* analysis revealed that the promoter region of *SFRP4* gene in diffuse astrocytomas was significantly more frequently methylated than in glioblastomas (*p* < 0.001), consistently glioblastomas had a significantly higher number of unmethylated promoters compared to diffuse astrocytomas (*p* < 0.001). Kruskal - Wallis test also confirmed significant differences in the methylation pattern within different astrocytoma grades (*λ* = 22.149; *p* < 0.001). The Mann–Whitney U test found that diffuse astrocytomas had significantly more methylated samples compared to anaplastic astrocytomas (*p* = 0.004) and glioblastomas (*p* < 0.001).

In addition, student’s *t*-test showed a significant connection between the age of tumor occurrence and the methylation of *SFRP4* gene promoters (*p* = 0.011). Younger patients were more likely to have a methylated promoter of the *SFRP4*. The mean age of patients with unmethylated promoters was higher (52.37 years) compared to age of patients with methylated promoters (41.5 years). A significant connection between the age of tumor occurrence and the methylation of SFRP4 gene promoters (*p* = 0.011) could also be influenced by the fact that in diffuse astrocytomas the age of onset is earlier than for glioblastomas.

Results of the MS-PCR reaction showed the presence of *SFRP4* promoter methylation in the majority of diffuse astrocytoma samples and the absence of methylation in higher astrocytoma grades (Table 3; Figure 5).

**Figure 6:**
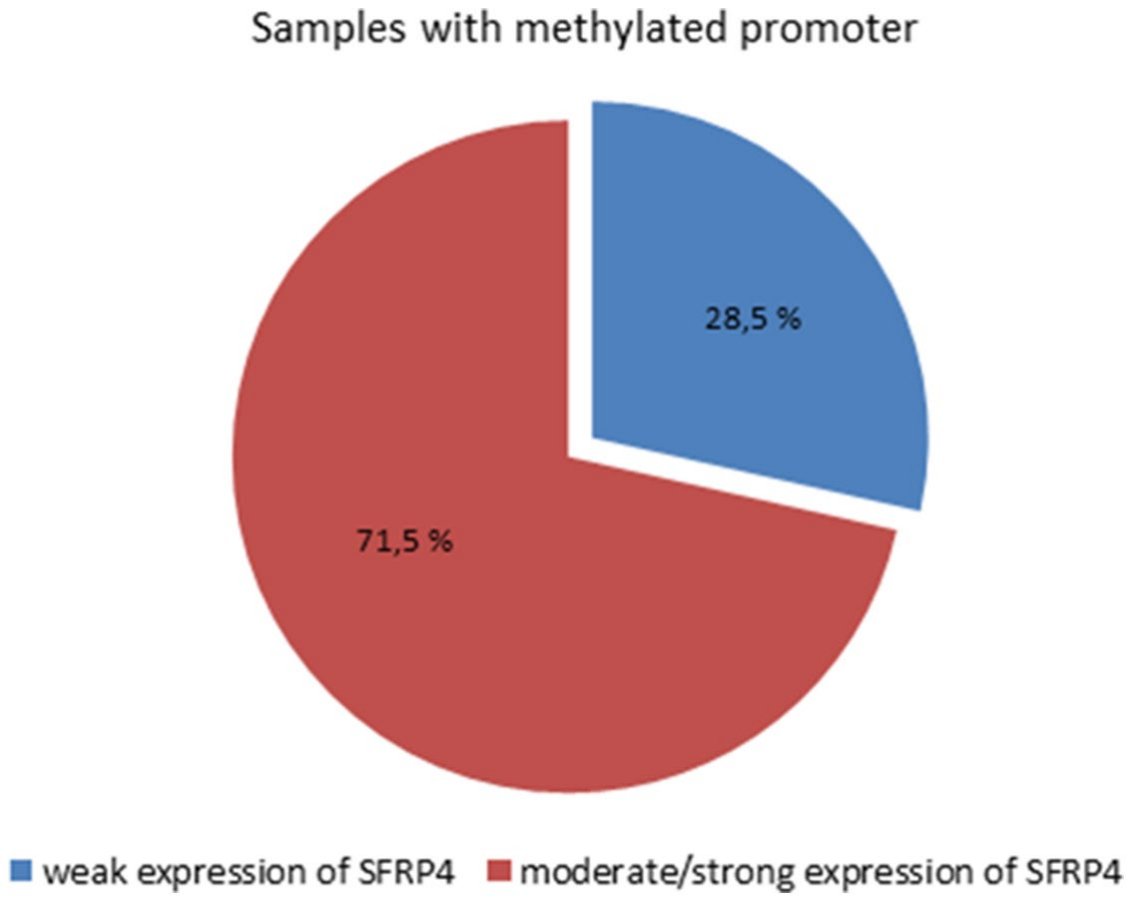
Connection between SFRP4 promoter methylation and protein expression.

The results of the χ2-test showed that there is a statistically significant connection between *SFRP4* gene methylation and SFRP4 protein expression (*p* = 0.007). Weak expression of the SFRP4 protein was observed in 28.5% of astrocytoma samples with methylated promoters and 78% of unmethylated samples. Moderate and strong SFRP4 expression was present in 71.5% of methylated samples and 22% of unmethylated samples (Figure 6). Furthermore, our results show that the expression levels of SFRP4 are strongly correlated with methylation of the *SFRP4* gene (Pearson’s R = − 0.413; *p* = 0.003). In the sense that, when the gene was methylated, protein levels were high, and when the expression was low or missing the gene was unmethylated. This behavior is contrary to the expected and indicates another mechanism besides methylation that turns off gene expression in higher grades.

Survival analysis revealed no significant impact of *SFRP4* promoter methylation nor SFRP4 protein expression on patients’ outcome.

### The correlations of SFRP4’s effect on GSK3β, DKK1, DKK3, LEF1 and β-catenin

We tested several players of Wnt signaling, namely GSK3β, DKK1, DKK3, LEF1 and β-catenin. We established a positive correlation between methylations of *SFRP4* and *GSK3β*. In samples where *SFRP4* was methylated, the same was observed for *GSK3β* (Pearson’s R = 0.323; *p* = 0.03). Additionally, when SFRP4 protein is expressed then *DKK3* gene was unmethylated (Chi square = 7.254; *p* = 0.027). Wnt signaling antagonist is associated to negative regulator’s demethylation. Correlations between SFRP4 and SFRP1, LEF1, DKK1, and β-catenin were not detected.

### *SFRP4* alterations from cBioPortal, COSMIC and LOVD

**Figure 7:**
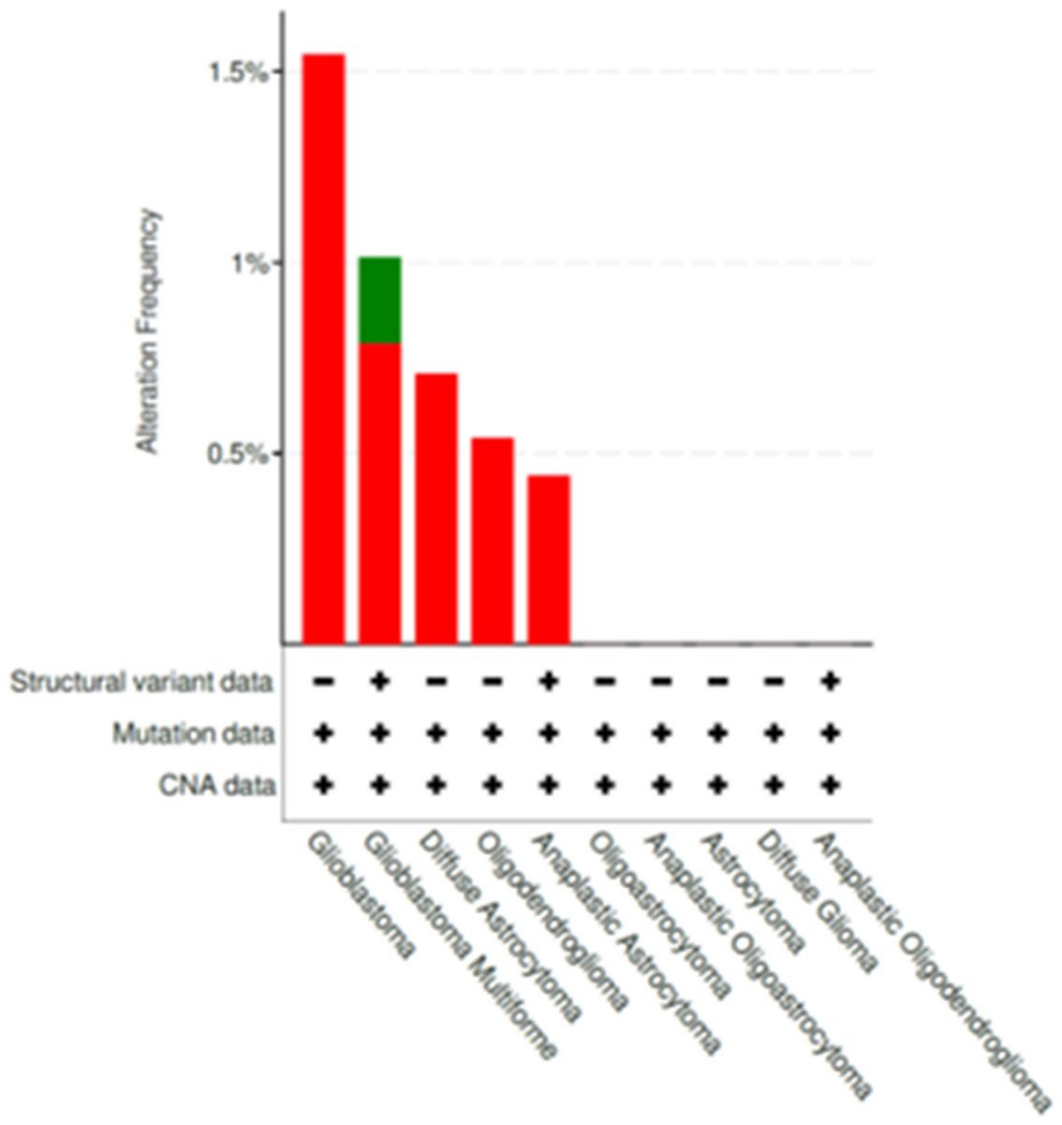
Changes publicly reported for SFRP4 gene from cBioPortal database (https://www.cbioportal.org/). Alterations that were listed for SFRP4 included amplifications and mutations.

**Figure 8:**
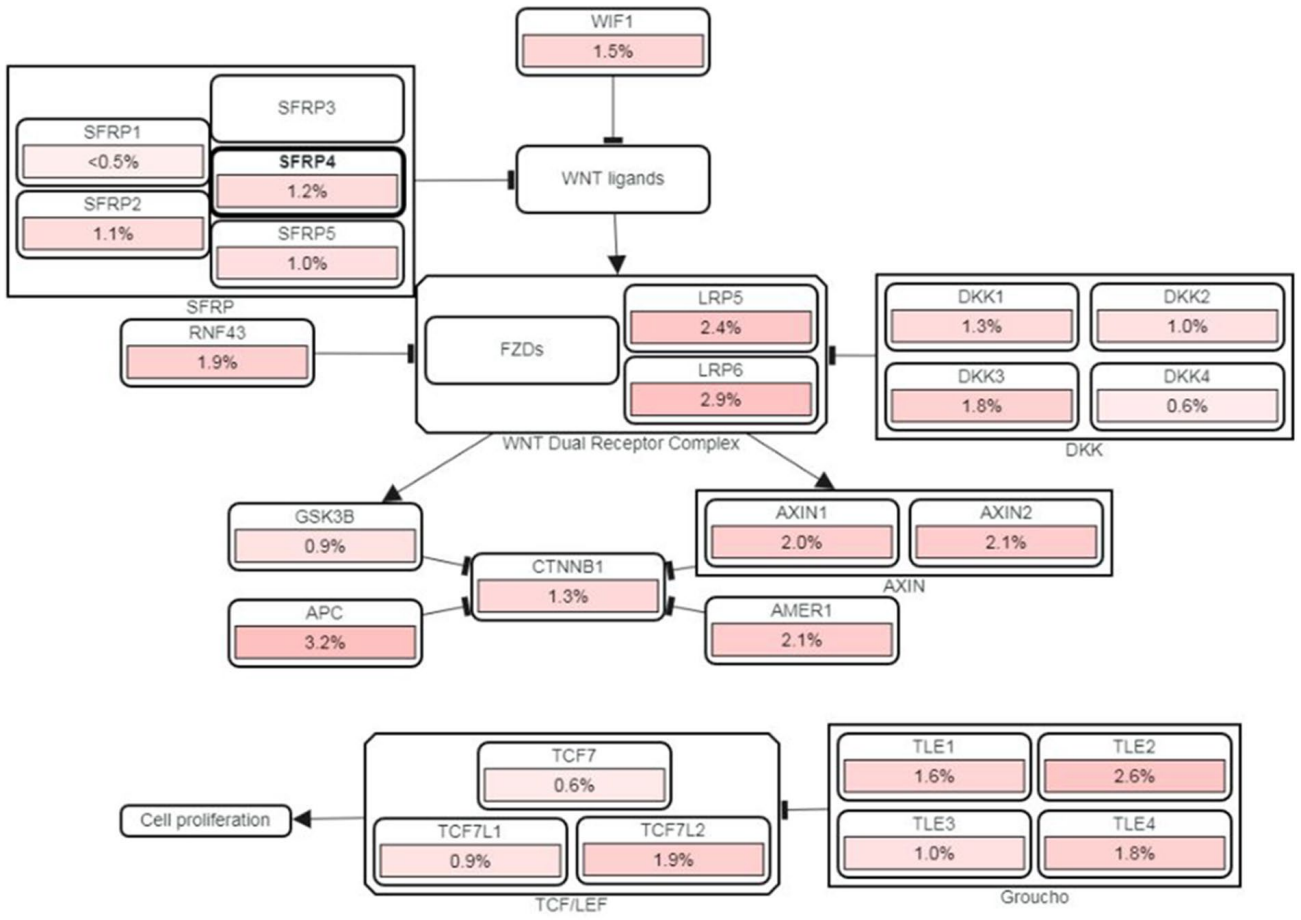
cBioPortal’s PathwayMapper graph showing percentage of changes of SFRP4 (circled bold) and of other components of Wnt signaling. The intensity of pink shades corresponds to a higher percentage of alteration.

The variants listed in cBioPortal and LOVD were rather scarce and can be viewed at the following URLs: https://www.cbioportal.org/results/mutations?cancer_study_list=lgg_tcga%2Cdifg_glass%2Cdifg_glass_2019%2Cdifg_msk_2023%2Cglioma_mskcc_2019%2Cglioma_msk_2018%2Clgg_ucsf_2014%2Cgbm_mayo_pdx_sarkaria_2019%2Cgbm_columbia_2019%2Cgbm_tcga_pan_can_atlas_2018%2Cgbm_cptac_2021&Z_SCORE_THRESHOLD=2.0&RPPA_SCORE_THRESHOLD=2.0&profileFilter=mutations%2Cstructural_variants%2Cgistic%2Ccna&case_set_id=all&gene_list=SFRP4&geneset_list=%20&tab_index=tab_visualize&Action=Submit; https://databases.lovd.nl/shared/users/03344, respectively. cBioPortal reports only two mutations in TCGA Pancancer Atlas: mutations S242F and C97S. Both mutations were found in glioblastoma and represent missense which are of unknown significance. For C97S PolyPhen, CADD, REVEL, MetaLR all predict to be likely deleterious. On the other hand, cBioPortal also reports on amplification of this gene (Figure 7). The percentage of changes in relation to other Wnt signaling components is shown in Figure 8. COSMIC reports on 6 coding regions mutations of SFRP4 in glioblastoma and one in anaplastic astrocytoma of which 5 are missense and two are silent ones and can be viewed on https://cancer.sanger.ac.uk/cosmic/gene/samples?all_data=&coords=AA%3AAA&dr=&end=347&gd=&id=373763&ln=SFRP4&seqlen=347&sn=central_nervous_system&src=gene&start=1#complete. The following mutations were reported: c.767 T > G, p.I256S, COSM9220797; c.737C > T, p.P246L, COSM9199206; c.677C > T, p.S226F, COSM9199207; c.532 T > C, p.C178R, COSM9199208; c.290G > C, p.C97S, COSM7481169; c.258C > T, p.T86=, COSM8264978; c.243C > T, p.Y81=, COSM8259479.

We also checked cBioPortal for survival and it demonstrated that patients with changed *SFRP4* had shorter survival than those without gene alterations.

## Discussion

Patterns of DNA methylation are changed in human malignant tumors. These pervasive changes encompass global hypomethylation of tumor genome but also the focal hypermethylation of numerous 5′-cytosine-phosphate-guanine-3′ (CpG) islands. The majority of CpG islands reside within or in close proximity to gene promoters. Their hypermethylation represents one of the mechanisms for gene silencing, which predisposes cells to malignant transformation (Bovolenta et al., 2008; Pawar and Rao, 2018). However, it has been challenging to associate specific DNA methylation changes in a cause-and-effect relationship for every step of tumorigenesis.

Functionally, Wnt antagonists can be divided into two classes. The first class binds the Wnt ligands and frizzled receptors directly and includes SFRP protein family, Cerberus and WIF-1 (Wnt inhibitory factor-1). The second class binds to LRP5/6 and includes the Dickkopf (DKK) family. In various tumors including gliomas, SFRP genes have been shown to be transcriptionally silenced by hypermethylation of the promoter region. Furthermore, SFRP proteins showed the ability to sensitize glioma cells to chemotherapeutic agents, cisplatin and doxorubicin, lower their proliferation rate, and induce apoptosis (Warrier et al., 2013). SFRP4 is a member of Wnt inhibitors that binds directly to Wnt and antagonizes Wnt pathways. Antiproliferative and proapoptotic roles for SFRP4 have been demonstrated during normal homeostasis in tissues such as ovary, corpus luteum, placenta, and mammary gland. Silencing of *SFRP4* gene in pathological states results in the activation of Wnt signaling which in addition to promoting tumor evolution also leads to the inhibition of apoptosis of tumor cells. Multiple different carcinoma cell lines that were transfected with recombinant SFRP4 demonstrated increased sensitivity to chemotherapeutics, decreased aggressiveness and invasiveness (Warrier et al., 2013).

Our study demonstrated that the protein levels of this Wnt antagonist were generally low with only 6% of our total astrocytoma sample showing strong expression of SFRP4 protein. Low SFRP4 expression predominated in higher astrocytoma grades (3 and 4) (*p* = 0.002) where 60% of grade 3 astrocytomas and 86.7% of glioblastomas showed weak or lack of expression. In the group of diffuse astrocytomas (grade 2) a significantly higher number of cases with moderate and strong expression was observed, 40% with moderate and 20% with strong expression. A significant difference between expression levels of SFRP4 protein regarding tumor grade (*p* = 0.008) was established. The increase in astrocytoma grade leads to a decrease in SFRP4 protein expression suggesting that SFRP4 acts as a tumor suppressor and inhibits the activity of the Wnt signaling. Similarly, Hrzenjak et al. (2004) showed a reduced expression of SFRP4 protein in more aggressive forms of endometrial sarcomas compared to lower-grades. The association of loss or reduced expression of SFRP4 protein with tumor progression has been documented in esophageal adenocarcinoma (Zou et al., 2005), pancreatic cancer (Bu et al., 2008), mesothelioma (He et al., 2005) and pituitary adenoma (Wu et al., 2015). These findings are in accordance with the results of our research. Conversely, there are other studies that have yielded controversial results on positive correlation between SFRP4 protein expression and tumor malignancy (Abu-Jawdeh et al., 1999; Mii and Taira, 2011; Xavier et al., 2014). Liang et al. (2019) which showed that nuclear SFRP3 and SFRP4 enhance the recruitment of β-catenin to the transcription factor TCF4, promoting transcriptional activity which also contributes to the tumor stemness. This result points on SFRP4 potential pro-oncogenic effect in some tumors which supports the claim on its dual role in tumorigenesis.

High frequency of DNA methylation at the CpG islands of the promoter regions of tumor suppressor genes is a common feature in human tumors and may occur at different stages of tumor evolution (Bovolenta et al., 2008; Pawar and Rao, 2018). Our analysis of methylation status of *SFRP4* gene across astrocytoma grades showed that the majority of samples (83.7%) did not have methylated promoter. Interestingly, *SFRP4* promoter methylation was exclusively observed in 72.7% of grade 2 astrocytoma, while in higher tumor grades methylated promoters were not detected. Statistical analysis revealed significant differences in the methylation status of the *SFRP4* gene between malignancy grades (*p* < 0.001). Astrocytomas grade 2 were significantly more methylated compared to astrocytomas grade 3 (*p* = 0.004) and glioblastomas (*p* < 0.001). All samples in which methylated promoters were detected, also showed bands denoting unmethylated promoters. Possible explanation of our result on methylation that is confined only to lower grade 2 is that in higher astrocytoma grades demethylation processes may occur. It is necessary to keep in mind that astocytomas harbor great heterogeneity and the tumor can consist of heterogeneous cells - some harboring methylated and some unmethylated promoters of *SFRP4* gene. It is possible that astrocytoma samples which did not show SFRP4 promoter methylation are regulated by alternative epigenetic regulatory events. Downregulation of sFRP4 expression in breast, prostate, and ovary cancer stem cells can be attributed to aberrant promoter hypermethylation together with histone modification (Deshmukh et al., 2019). Furthermore, Belur Nagaraj et al. (2021) identified miR-181a as activator of Wnt/β-catenin signaling that drives stemness and chemoresistance in ovarian cancer via the inhibition of SFRP4. MicroRNA-96-5p facilitated the viability, migration, and invasion of cervical cancer cells by silencing SFRP4 (Zhang et al., 2020).

Novel findings report that specific types of tumors, such as low-grade gliomas, are characterized by a so-called CpG island methylator phenotype (CIMP) which could explain our results. CIMP phenotype is characterized by few thousand CpG islands that are methylated simultaneously in an individual cancer sample (Pfeifer, 2018; Malta et al., 2024) and need not to be confined to promoter region. We can speculate that methylation of *SFRP4* confined only to grade 2 astrocytomas may act as “driver methylation” that initially inactivates relevant suppressor gene (Pfeifer, 2018). CpG sites are dispersed throughout the genome and are usually methylated, called CpG islands (CGIs). CGIs located at promoter regions are generally unmethylated. Methylomes contain parts of DNA containing frequent CpG sites, CGIs usually overlap gene promoters and are located at the 5′ end of genes. However, they can be located in gene bodies and in other regions, too. So, we have CpG shores (2 kb regions flanking CGIs), CpG shelves (>2 kb regions flanking CpG shores), and also open sea regions (>4 kb to the nearest CGIs). It is obvious that methylomes are versatile in physiological circumstances. In cancer, DNA methylation becomes aberrant mostly by focally hypermethylating promoters of genes but also gene bodies (Pfeifer, 2018; Malta et al., 2024).

Several different studies reported hypermethylation of the *SFRP4* gene promoter and decreased expression of the SFRP4 protein in tumors of the endometrium, cervix, bladder, pancreas, kidney, esophagus, pituitary gland, and mesothelioma (Pohl et al., 2015). Meta-analysis by Yu et al. (2019) revealed an increased risk of colorectal, ovarian, cervical and kidney cancers associated with methylation of the *SFRP4* promoter, while no such risk was found for endometrial and stomach cancers. Nevertheless, a large heterogeneity within the groups was observed in this meta-analysis. Investigations on four different human glioblastoma cell lines by Schiefer et al. (2014) showed that *SFRP4* gene silencing induced by promoter methylation is one of the glioblastoma features. The four remaining SFRPs were also hypermethylated in all four glioblastoma cell lines. This is contrary to our results on 16.3% of methylated promoters confined only to the lower grade.

In our cohort of astrocytomas, glioblastomas were found to occur later in life compared to grade 2 (*p* = 0.003) and grade 3 cases (*p* = 0.027). It has been shown previously that epigenetic changes and mutation frequencies are distinct between primary and secondary glioblastomas (Barthel et al., 2018). GBM have traditionally been divided into primary (accounting for 90% of cases and arising *de novo*) and secondary (accounting for 10% of cases and developing from a pre-existing lower grade tumor) (Ohgaki and Kleihues, 2013). These historical terms now correlate closely to IDH-mutation status: primary or *de novo* GBMs are classified as IDH-wildtype GBMs. In contrast, IDH-mutant GBMs are defined as secondary GBMs and are currently included in astrocytoma WHO CNS grade 4 (Ohgaki and Kleihues, 2013; Louis et al., 2016). We have also demonstrated here that *SFRP4* promoter methylation was more frequent in younger patients (*p* = 0.011). One possible explanation is that CNS grade 2 and grade 3 astrocytomas coincide to younger age. Also, it has been demonstrated that the process of ageing contributes to the general loss of methyl groups (Unnikrishnan et al., 2019). Foltz et al. (2010) indicated that posttranslational modifications of histones are responsible for the modulation of Wnt pathway antagonists. Götze et al. (2010) investigated 70 astrocytic gliomas for promoter hypermethylation of different Wnt pathway inhibitor genes including *SFRP4*. Results revealed that hypermethylation of *SFRP4* was rare in gliomas, in only 6% of tumors. The cutting-edge research proposes that DNA methylation profiling is indispensable for identification of specific tumor types (Malta et al., 2024). DNA methylation profiling continues to identify numerous tumor types with specific methylation patterns that have characteristic genetic alterations and clinical behavior. Glioma epigenome remains incompletely characterized especially its effect on progression and recurrence. Molecular changes at other levels and other genes in concert with epigenetics, especially methylation, are also not adequately characterized. Therefore, unique DNA methylation profiles could be very helpful in diagnosis of specific subtypes of diffuse gliomas. Glioma Longitudinal AnalySiS (GLASS) international consortium (Malta et al., 2024), analyzed an epigenetic cohort of glioma patients with matched initial and first recurrent tumors and showed that IDHwt gliomas have lower DNA methylation levels with aggressive cases having the lowest genome-wide levels of DNA methylation. Even within IDH-mutant gliomas, a subset of cases presented with a lower degree of DNA methylation had poorer outcome, while highly methylated ones had better prognosis (Malta et al., 2024).

Our finding on significant connection between *SFRP4* gene methylation and its protein expression (*p* = 0.007) backed up with strong negative correlation (Pearson’s R = − 0.413; *p* = 0.003) can be interpreted that when the gene was methylated, protein levels were high, and in turn when the levels were low or missing, the gene was unmethylated. This behavior is contrary to the expected and suggests that the expression of *SFRP4* gene in higher grades has been regulated by alternative mechanisms. It is possible that *SFRP4* is targeted by genetic alterations as it has been described for *SFRP1* in colon cancers. Other possible epigenetic mechanisms may also be involved. An equally important mechanism to silence genes, besides DNA methylation, are genetic alterations. Although our study did not examine alternative mechanisms of *SFRP4* silencing our search through the public databases revealed four missense mutations found in glioblastoma and one in anaplastic astrocytoma. Several mutations of SFRP4 gene are reported as part of Pyle disease etiology and altogether 105 mutations can be found in different cancers,^2^ interestingly the ones that have been characterized as pathogenic all result in frameshift or nonsense indicating that the protein is lost. It is important to stress that the mutational profile availability was not very frequently reported. There are only few reported mutations of SFRP4 gene throughout the analyzed databases, cBioPortal, COSMIC and LOVD. A study by Liu et al. (2006) suggests that silencing of SFRPs by CpG island methylation is involved in chronic lymphocytic leukemia (CLL) as one possible mechanism contributing to aberrant activation of Wnt pathway. Marked differences in the levels of aberrant DNA methylation between *SFRP* genes was found, namely, *SFRP1* was methylated in 100% of cases, *SFRP2* in 55%, *SFRP4* in 30%, and *SFRP5* in 15%, suggesting that epigenetic silencing of these SFRPs and especially *SFRP1* could be important in the onset of CLL. However, *SFRP2* and *SFRP4* were also frequently silenced in CLL, although not through CpG island methylation. SFRP4 was downregulated or silenced in 9.1% of colorectal adenomas relative to the normal mucosa. The downregulation of SFRP 2, 4 and 5 was more frequent in colon carcinoma than in adenoma. However, the methylation and downregulation of SFRP4 were less common than SFRP1, 2 and 5 genes in colorectal tumor, though they were both high in mesothelioma and esophageal adenocarcinoma (Qi et al., 2006). Wallner et al. (2006) also showed that the frequency of methylated *SFRP4* gene in serum DNA of the metastasized colon cancer increased when compared to local disease.

Collective results of our previous work have shown that glioma proliferation and invasion are fueled by Wnt signaling activation (Pećina-Šlaus et al., 2014; Kafka et al., 2019). We have found that promoters of selected genes displayed different methylation frequencies (Pećina-Šlaus et al., 2011; Kafka et al., 2017, 2021). *DKK3* and *DKK1* displayed the highest methylation frequencies 43% and 38%, respectively. *SFRP1* followed with 32%, while *GSK3β* promoters were less methylated, in 18% of samples (Kafka et al., 2018). GSK3β (glycogen synthase kinase 3) is a key enzyme in Wnt signaling. Dickkopfs (DKK) act as inhibitors of the WNT pathway by binding to low-density lipoprotein receptor-related proteins (LRP) 5/6 and Kremen. DKK3 is omnipresent in normal human tissues, including the brain; however, it is significantly depleted in various cancer cell types. Additionally, we have demonstrated that the number of samples with hypermethylated promoter of *SFRP1* gene increased in glioblastomas (grade 4, *p* = 0.042) compared to lower grades, which is contrary to the present situation with *SFRP4*. Also contrary to the result of the present study, is the behavior of SFRP1. Samples with methylated promoter expressed significantly less protein than unmethylated ones (*p* = 0.031). Therefore, in the present investigation we decided to correlate SFRP4’s effect with the findings on GSK3β, DKK1, DKK3, LEF1 and β-catenin. A positive correlation between methylations of *SFRP4* and *GSK3β* genes was shown. In samples where *SFRP4* was methylated, the same was observed for *GSK3β* gene (Pearson’s R = 0.323; p = 0.03). Another positive correlation was observed between SFRP4 expression and *DKK3* methylation. When SFRP4 is expressed then *DKK3* gene was unmethylated (Chi square = 7.254; *p* = 0.027) indicating that Wnt signaling antagonist is associated to negative regulator’s demethylation.

And finally, it is important to mention glioma stem cells (GSCs) and Wnt antagonists. A previous study reported that Wnt antagonists are epigenetically silenced in glioblastoma. Furthermore, it has been reported that the SFRP4 reduces the stemness of glioblastoma by its netrin-like domain. The authors hypothesized that the Wnt pathway could be important in maintaining glioma stemness (Schiefer et al., 2014). This suggests that SFRP4 may have destructive effect on GSCs and holds potential as epigenetic-based therapy with demethylation agents.

Wnt signaling is being extensively investigated together with therapeutic strategies to target pathway components. SFRP4 is an interesting molecular factor in the occurrence and development of astrocytomas. Reduced expression or silencing of *SFRP4* gene results in overactivation of Wnt pathway. The results of present investigation show reduced expression of SFRP4 in glioblastomas compared to lower grade diffuse gliomas, indicating its tumor suppressor character. SFRP family members were originally considered inhibitors of Wnt signaling, however, it has been reported that SFRP4 has the least homology with other family members. Our results may suggest that the Wnt signaling is regulated by different SFRP molecules in different context. Additional research is indicated to determine the mechanism, genetic or epigenetic, that is behind SFRP4 lowered expression in higher astrocytoma grades. The limitation of this study is relatively small number of samples and the inability to reveal the whole methylation pattern of this gene. However, the study contributes to the recognition of the significance of epigenetic changes in diffuse glioma indicating that restoring SFRP4 protein holds potential as therapeutic avenue.

## Data availability statement

The original contributions presented in the study are included in the article/supplementary material, further inquiries can be directed to the corresponding author.

## Ethics statement

The studies involving humans were approved by Ethical Committees, School of Medicine University of Zagreb (Case number: 380-59-10106-14-55/147; Class: 641-01/14-02/01) and University Hospital Center “Zagreb” (number 02/21/JG, class: 8.1.-14/54-2). The studies were conducted in accordance with the local legislation and institutional requirements. The participants provided their written informed consent to participate in this study.

## Author contributions

AK: Conceptualization, Data curation, Formal analysis, Investigation, Methodology, Supervision, Validation, Writing – original draft. NP-Š: Conceptualization, Data curation, Formal analysis, Funding acquisition, Investigation, Project administration, Resources, Supervision, Writing – original draft. DD: Investigation, Methodology, Writing – original draft. AB: Formal analysis, Methodology, Validation, Writing – review & editing. NN: Data curation, Formal analysis, Validation, Writing – review & editing. KŽ: Data curation, Formal analysis, Validation, Writing – review & editing. AJ: Data curation, Formal analysis, Validation, Writing – review & editing.

## Footnotes

^1^ https://cancer.sanger.ac.uk/cosmic

^2^ https://www.ncbi.nlm.nih.gov/clinvar
